# Non-Arteritic Anterior Ischemic Optic Neuropathy (NA-AION): A Comprehensive Overview

**DOI:** 10.3390/vision7040072

**Published:** 2023-11-09

**Authors:** Maria Letizia Salvetat, Francesco Pellegrini, Leopoldo Spadea, Carlo Salati, Marco Zeppieri

**Affiliations:** 1 Department of Ophthalmology, Azienda Sanitaria Friuli Occidentale, 33170 Pordenone, Italy; mlsalvetat@hotmail.it (M.L.S.);; 2 Eye Clinic, Policlinico Umberto I, “Sapienza” University of Rome, 00142 Rome, Italy; 3 Department of Ophthalmology, University Hospital of Udine, 33100 Udine, Italy

**Keywords:** ischemic optic neuropathy (ION), non-arteritic anterior ischemic optic neuropathy (NA-AION), NA-AION pathophysiology, NA-AION risk factors, NA-AION treatment options

## Abstract

Non-arteritic anterior ischemic optic neuropathy (NA-AION) represents one of the most important causes of blindness or severely impaired vision in middle-aged and elderly people. Unilateral optic disc edema and abrupt, painless vision loss are its defining features. It is commonly assumed that NA-AION is caused by an ischemic infarction of the optic nerve head, and, although the exact pathogenesis is still unknown, several risk factors and comorbidities associated with its development have been found. NA-AION occurs generally in patients older than 50 years who have small optic discs and vasculopathy risk factors. Even though numerous treatment options have been proposed, no available effective medical or surgical therapy or prophylactic measure for NA-AION currently exists. The purpose of present-day therapeutic strategies is therefore to identify and possibly control any underlying modifiable risk factors, aiming to prevent the development of new NA-AION episodes in the affected and fellow eye. A thorough assessment of NAION, including its history, epidemiology, etiology, pathophysiology, risk factors, associated comorbidities, clinical findings, diagnostic tests, treatment choices, prognosis, and future research, is the goal of this work.

## 1. Introduction

Ischemic optic neuropathies (IONs) represent a major cause of blindness or severe impaired vision amongst middle-aged and elderly subjects and refer to a state of ischemic damage of the optic nerve (ON) [1,2,3,4,5,6].

From a clinical point of view, IONs can be divided into anterior (AION) and posterior forms (PION), and are clinically characterized by the presence or the absence of optic disc (OD) edema, respectively; furthermore, IONs are classified as arteritic (A-ION), if caused by arteritis, and non-arteritic (NA-ION) or idiopathic [1,2,3,4,5,6], the etiology and pathophysiology of which is still debated [7,8,9].

Non-arteritic anterior ischemic optic neuropathy (NA-AION) is a major cause of blindness or severely impaired vision in adults, representing the most common cause of acute optic neuritis in patients older than 50 years and the second most frequent form of all optic neuritis after glaucoma [10]. NA-AION is a medical condition that affects the optic nerve, leading to sudden and often painless vision loss. This condition typically occurs in one eye and is characterized by a disruption of blood flow to the optic nerve, which can result in damage and impaired vision. NA-AION is distinct from arteritic anterior ischemic optic neuropathy (AAION), which is associated with giant cell arteritis and can cause more severe vision loss and even blindness. NA-AION is most commonly observed in older individuals and is often associated with various risk factors such as hypertension, diabetes, and certain medications. Early diagnosis and management are crucial to mitigate vision loss in affected individuals.

An overview of NA-AION, including its epidemiology, etiology, clinical manifestation, diagnostic assessment, therapeutic choices, and prognosis, is the aim of this study. We discuss NA-AION’s historical notes as well as the state of our knowledge of its pathophysiology and risk factors. We also review the signs, symptoms, diagnostic tests, and clinical findings associated with NA-AION’s clinical presentation. We also present different available NA-AION treatments, including both medical and surgical alternatives, as well as the outlook for NA-AION patients. Finally, we highlight new lines of inquiry that will help with NA-AION care, diagnosis, and prevention.

## 2. History and Terminology

The term “ischemic optic neuropathy” has been used in the medical literature for many years to describe conditions involving inadequate blood supply to the optic nerve, leading to vision loss. “Non-arteritic” in NA-AION refers to the fact that this condition is not associated with inflammation of the arteries (as in arteritic anterior ischemic optic neuropathy or AAION). “Anterior” indicates that the damage occurs in the front portion of the optic nerve, closer to the eye. “Ischemic” underscores the central mechanism of NA-AION, which involves a restriction of blood flow to the optic nerve. “Optic neuropathy” denotes the involvement of the optic nerve, which connects the eye to the brain and is crucial for vision. Understanding the historical context and the terminology surrounding NA-AION is essential to understand its development as a distinct clinical entity and its place within the broader field of ophthalmology and neurology.

The French physician Jean-Pierre Saint-Yves first documented NA-AION instances at the beginning of the 19th century, in 1817. The disorder was not fully understood until ophthalmologists started to describe it in greater depth in the 20th century. NA-AION was given a thorough clinical description by C. Miller Fisher in 1935, who emphasized the condition’s quick onset, lack of pain, and connection to OD edema. The underlying pathophysiology of NA-AION, which involves ischemic injury to the optic nerve head (ONH), has since been clarified thanks to improvements in imaging and diagnostic methods but, despite these developments, the precise cause of NA-AION remains unclear [2,3,5,8,9]. Dr. Sanders reported NA-AION cases in 1963, although the term “anterior ischemic optic neuropathy” was not coined until the early 1970s by Dr. Sohan Singh Hayreh [7]. The research revealed that the disease was a separate entity from giant cell arteritis, which was first assumed to be related to it, so the definition of “non-arteritic anterior ischemic optic neuropathy” was introduced [11].

## 3. Epidemiology

NA-AION is one of the leading causes of sudden, painless vision loss in adults over the age of 50. NA-AION is the most prevalent acute optic neuropathy in middle-aged and elderly people, with an estimated annual incidence rate of 2.3 to 10.2 per 100,000 people in the United States [10]. The condition is more prevalent in individuals of European descent, but it can affect people of all races and ethnicities. Caucasians experience the illness more frequently than people from other ethnic groups [12]. The incidence in Black and Asian populations seems to be lower [10,13].

Although previous studies did not find any gender predisposition [10], an association with the male gender has been reported by other authors [12,14], especially in Asian populations [13].

NA-AION generally affects patients over 50 years of age, with the frequency rising with advancing years [10]; however, the incidence in patients younger than 50 years is not rare and has increased in recent years [15].

Various systemic risk factors are associated with NA-AION, including hypertension, diabetes, sleep apnea, and certain medication use (such as Viagra). In many cases, NA-AION occurs unilaterally (in one eye), but it can, rarely, affect both eyes sequentially. Despite being relatively common, the exact prevalence and incidence of NA-AION can vary by region and may be underreported since not all affected individuals seek medical attention.

Understanding the epidemiological aspects of NA-AION is crucial for healthcare providers and researchers to better comprehend its occurrence, risk factors, and potential strategies for its prevention and management.

## 4. Etiology and Pathophysiology

The exact etiology and pathophysiology of NA-AION are still highly controversial [3,5,9], and the paucity of histologically examined NA-AION cases contributes to an incomplete understanding of its pathogenesis [16]. However, it is commonly accepted that NA-AION is caused by an acute circulatory insufficiency of the OHN, i.e., a hypo-perfusion or hypo-ossigenation, resulting in an acute ischemic infarction of the ON axons, and secondary apoptosis of the retinal ganglion cells (RGCs) [3,5,7,8,9,17].

The primary cause of NA-AION is a compromised blood supply to the optic nerve, which leads to ischemia (inadequate blood flow). The exact etiology of NA-AION is multifactorial, but several systemic and local factors can contribute to its development. Risk factors for NA-AION include conditions such as hypertension, diabetes, sleep apnea, atherosclerosis, and certain medications. Smoking and cardiovascular diseases can also increase the risk.

Several precipitating factors have been suggested to explain the occurrence of an acute deficit in ONH blood-flow supply in NA-AION, including generalized hypo-perfusion, nocturnal hypotension, vasospasm, local arteriosclerosis vascular occlusion, venous occlusion, thrombosis, embolization, or multiple micro-embolisms from a remote source, even if a precise cause is not found in the majority of cases [7,8,9,17].

Moreover, considering that studies in healthy subjects have demonstrated that ONH blood flow is maintained relatively constant despite ocular perfusion pressure level changes by vascular autoregulation mechanisms [18], it has been suggested that impaired ONH blood-flow autoregulation, or precipitating factors that overcome its operative range, may play an important role in NA-AION development [1,7].

NA-AION typically occurs when there is a disruption in the perfusion of the posterior ciliary arteries that supply blood to the optic nerve head (optic disc). This reduced blood flow results in hypoxia (oxygen deprivation) and insufficient nutrient supply to the optic nerve, leading to damage. The exact sequence of events within the optic nerve is complex, involving inflammation, swelling, and, ultimately, axonal damage and loss of retinal ganglion cells. Optic nerve head edema, often observed during the acute phase, can lead to the characteristic optic disc swelling (disc edema) seen in NA-AION. Over time, the disc edema may resolve, leaving optic disc atrophy and visual field defects, which are hallmarks of NA-AION.

Insights into ON vascularization, the events related to ischemic injuries of the neural tissues, and the assumed NA-AION pathogenetic sequence are presented below.

### 4.1. The Optic Nerve Vascularization

The ON, formed by the RGCs axons, is composed of four parts: the intraocular portion, also called ONH or OD, the intraorbital, intracanalicular, and intracranial portions. The ONH can be divided into three anatomically distinct zones: the prelaminar or anterior portion, the lamina cribrosa or middle portion, and the retrolaminar or posterior portion [17].

The ONH is vascularized by branches of the ophthalmic artery, which represent the terminal portion of the internal carotid artery, named short posterior ciliary (SPC) arteries. The SPC arteries arise from the ophthalmic artery when it crosses the ON and divide into 10–20 branches. The ONH prelaminar region is supplied by the SPC arteries and from capillaries derived from the retinal circulation; the lamina cribrosa is vascularized by the SPC arteries either directly or by forming the Zinn and Aller’s circle; and the retrolaminar region is supplied by the pial vessels perforating the ON surface [19]. Several interactions between the vascular supply of the laminar, pre-, and retro-laminar regions exist [19]. The other ON portions receive blood supply from the pial circulation and capillaries arising from the ophthalmic artery [19].

Although several instruments can provide an estimation of ONH blood flow in vivo, including fluorescein angiography (FAG), indocyanine green angiography (ICG), optical coherence tomography angiography (OCT-A), laser speckle flowgraphy, and laser Doppler flowmetry [20,21], reliable clinical methods to measure the exact amount of ONH perfusion in vivo are not yet available [19].

ONH blood flow is assumed to be directly related to the ocular perfusion pressure (OPP), defined as the difference between the mean systemic arterial blood pressure and intraocular pressure (IOP) [22]. By definition, both systemic hypertension and IOP reduction may cause an increased OPP, i.e., an ONH blood-flow enhancement; on the other hand, systemic hypotension or ocular hypertension could induce ONH blood-supply impairment [23].

In physiological conditions, in the presence of systemic arterial blood pressure and IOP modifications, ONH and retina blood flow is maintained relatively constant by vascular autoregulation mechanisms that are mainly related to changes in the smooth muscle tone of the terminal arterioles and the contractile activity of the pericytes of the capillaries [1,17,18]. Although the exact mechanism of blood-flow autoregulation is still unknown, the release of various substances by the vascular endothelium, including endothelin-1 and nitric oxide, is thought to play a fundamental role [18]. Moreover, animal studies have demonstrated that autoregulation operates only over a critical range of OPP, which in monkeys has been estimated to be ≥30 mmHg: with OPP under this threshold, the autoregulation becomes ineffective, so that ONH blood flow is directly proportional to the OPP [1]. It is supposed that this scenario could be valid also in humans, even if a clinical method to evaluate autoregulation in healthy subjects and patients is not yet available [1]. Finally, several factors may disrupt these autoregulatory mechanisms, including aging, arterial hyper- and hypotension, diabetes, hyperlipidemia, thyroid disease, cerebrovascular diseases, OSAS, and vasoactive drugs, etc. [1,17,18]. An altered vascular autoregulation may increase ONH sensitivity to minor IOP or arterial blood pressure changes, with an increased risk of ONH ischemia development [17].

### 4.2. The Hypothesized Pathogenetic Sequence in NA-AION

The hypothesized cascade of events in NA-AION may be summarized as follows [7,8,9,17]:

***a. Initial transitory hypoperfusion event of the OHN.*** The data supporting this assumption can be summarized as follows:

- histological specimens of rare human clinically proven acute NA-AION cases have shown an ischemic infarction of the retrolaminar part of the ONH, with variable involvement of the laminar and prelaminar regions [24]. The OD ischemic edema was not distributed following the watershed zones [24,25,26]; moreover, the SPC arteries and their tributaries showed only age-related changes, without a clear mechanical occlusion due to emboli or thrombosis [24,26]. These data suggest that an acute transient non-perfusion or hypoperfusion of the SPC arteries, followed by reperfusion, could likely be the most frequent NA-AION pathogenetic mechanism [16]. However, Dr. Sohan Singh Hayreh has described rare cases of thrombo-embolic occlusion of the SPC arteries in NA-AION patients [7,11]. Compared to the hypotensive form of NA-AION, the thromboembolic type has been associated with more severe ONH damage and a worse final visual prognosis [7,11];
- OD and peripapillary choroid ischemia have been demonstrated in NA-AION patients during the acute phase of the disease by several methods:
  - fluorescein angiography (FAG) images have shown signs of total or partial prelaminar OD ischemia (55–75% of cases) and, less frequently, of peripapillary choroid ischemia (25% of cases). The ischemic insult could have varying degrees of severity, ranging from mild perfusion delay to severe impaired perfusion [11,15,27]. Moreover, the fluorangiographic filling delay did not follow a watershed distribution [27], suggesting that microcirculatory impairment in NA-AION eyes should affect the paraoptic branches or their tributaries within the disc and not the SPC arteries [27]. FAG signs of ischemia are typically absent in non-ischemic OD swelling [28], suggesting that vascular insufficiency may be the cause rather than a secondary effect of the OD edema;
  - indocyanine green angiography (ICG) [29] and laser speckle flowgraphy [30] studies have documented a decrease in OD and peripapillary perfusion;
  - optical coherence tomography (OCT)–angiography images have demonstrated a significant segmental or global reduction in OD and peripapillary region vessel density in eyes affected by acute and chronic NA-AION in comparison with healthy eyes [31];

All these data support a role for ischemia in the pathophysiology of NA-AION, although the precise site of the vessel occlusion has not been proven [16].

***b. Early disruption of the ONH blood-brain barrier, with increased capillary permeability causing OD edema that develops before the clinical symptoms.*** This supposition is supported by the following data:

- the presence of dye leakage in the FAG images: the characteristic FAG features in acute NA-AION cases include early OD segmental hyperfluorescence and late OD leakage (50–70% of cases) [11,15,27], consistent with increased ONH capillary permeability;
- the existence of an “incipient pre-clinical NA-AION”, a clinical entity characterized by an asymptomatic OD edema that can spontaneously resolve, which is explained as a reversible ONH ischemia without infarction, or progress to an overt NA-AION within a few weeks (25–45% of cases) [32]. A pre-symptomatic phase of NA-AION with impaired perfusion of the ONH has been confirmed by fluorescein angiography [15];

***c. Successive ON damage from a “compartment syndrome”***: it is thought that the edema developing in a restricted and non-expansible area, such as that extending between the ONH surface and the lamina cribrosa, can increase ONH damage by compressing the following elements: ON axons, with axoplasmic flow blockage; capillaries and arterial vessels, with secondary ischemia of axons and glia tissue; and venous vessels, with increased OD edema (vicious circle). The theory of the “compartment syndrome” is sustained by the following observations:

- human histopathological studies in acute NA-AION cases have shown that the ONH axon ischemia does not follow a specific vascular pattern, suggesting that an axonal and vascular compression may be responsible for the majority of the damage, without respect for the vascular territories [24,26];
- a crowded OD, i.e., an OD with a small diameter and small cup-to-disc ratio (C/D) (OD diameter < 1.5 mm, C/D < 0.2), also indicated as “disk-at-risk”, is considered to be a predisposing or contributing factor in the development of NA-AION [33], and it has been found in the fellow eye in approximately 80% of NA-AION patients [34], especially in those younger than 50 years [15];
- OD drusen, formed by aggregates of calcified materials accumulating as a consequence of axoplasmic transport alterations at the ONH, may induce exaggerated OD crowding and has been associated with the occurrence of NA-AION, especially in patients younger than 50 years [15,35,36];
- a recent prospective, comparative OCT study has demonstrated that, as compared to healthy controls, patients affected by NA-AION have increased prelaminar thickness and peripapillary choroidal thickness in both the affected and unaffected eye [37], supporting the hypothesis that the best theatre for NA-AION development seems to be a restricted and inextensible region;

***d. Secondary inflammation at the site of ischemic insult,*** which may contribute to axon and capillary endothelial cell damage. The role of inflammation secondary to the ONH ischemia in increasing axon damage and visual loss is supported by the following observations:

- in vitro and animal model studies investigating the cellular mechanisms of the ON axons and RGCs ischemic damage have demonstrated that the dying cells induce a toxic environment by releasing glutamate, and reactive oxygen species, with further cell damage, apoptosis, and death of the surrounding tissue, suggesting that, after the initial phases, the cell damage could be related to inflammation rather than to ischemia [38];
- a neutrophil-mediated cellular inflammation response, typically found in areas of ischemic injury, has been demonstrated in experimental animal models of acute NA-AION at the site of the ONH infarction [38];
- ON specimens obtained from patients with a well-documented NA-AION in acute or chronic phases showed macrophage-based inflammation at the ONH [24,25,26];
- recent clinical studies have demonstrated an association between NA-AION and increased levels of serum inflammation markers, in particular, an increased neutrophil-to-lymphocyte ratio, platelet count, and systemic immune–inflammation index [39].

These data may indicate that inflammation may play a role in the pathogenesis of NA-AION and suggest that selective inflammatory response modulation may be an approach in the treatment of NA-AION [39];

***e. Final axons and glia necrosis and RGCs death by apoptosis.*** Previous studies have shown the following findings:

- NA-AION animal models of both rodents and non-human primates, that utilize laser light to activate intravascular photoactive dye to induce capillary vascular thrombosis, have documented the final occurrence of ON axon loss and RGCs death by apoptosis [40]. Ischemia-induced RGC death seems to be mainly related to neurotrophin deprivation [40];
- human histopathological studies of acute NA-AION cases have shown coagulative necrosis of ONH axons and glial cells; in the chronic NA-AION phase, a severe axonal loss was histologically demonstrated [24,25,26];
- using OCT images, several authors have shown that, in comparison to healthy eyes, those affected by NA-AION in the chronic phase showed a significant reduction in peripapillary retinal nerve fiber layer (RNFL) thickness due to the evolution towards optic atrophy, and a significant reduction in the macular ganglion cell layers caused by retrograde maculopathy [41,42,43].

## 5. Risk Factors and Associated Comorbidities

NA-AION is considered to be a multifactorial disorder in which the causative event, i.e., the acute ischemia of the ONH, could be caused by different combinations of local and systemic risk factors in different patients. It has been calculated that more than 70% of NA-AION patients had pre-existing risk factors and comorbidities that can deeply impact prognosis and treatment options [12,14,44]. The prevention of NA-AION and better patient outcomes is commonly thought to be related to early diagnosis and prompt treatment of these conditions [3,5,11,45].

Risk factors for NA-AION development, theoretically divided into predisposing or precipitating [11], and further classified into “anatomical variations, pharmaceutical use, and systemic vascular risk factors”, include:

- ***Older age***: NA-AION generally affects patients over 50 years of age, with an onset age ranging between 57 and 65 years [10]. Approximately 7.5–25% of NA-AION patients are younger than 50 years [15]. The so-called “NA-AION of the young” seems to be more frequent in Asian populations [13];
- ***Male gender***: although previous studies did not find gender differences [34], recent large scale meta-analyses found an association between male gender and NA-AION [14], especially in Asian populations [13];
- ***Caucasian race***: 95% of NA-AION patients in the United States are Caucasian [12]; the reported incidence in Black and Asian populations is statistically lower [10,13]. In particular, Black populations could be less affected by the disease because of their tendency to have large C/Ds, whereas a crowded disc is one of the most important risk factors for NA-AION development [12,14];
- ***Crowded disc***: a crowded OD, i.e., an OD with a small diameter and small cup-to-disc ratio (C/D) (OD diameter < 1.5 mm, C/D < 0.2), also indicated as “disk-at-risk”, has been found in the fellow eye in 80–90% of NA-AION patients [33,34], with higher percentages in patients younger than 50 years [15]. A crowded OD is considered to be a predisposing or contributing factor rather than a primary causative factor in the pathogenesis of NA-AION. It is indeed supposed that localized swelling occurring in a small and crowded OD, especially in the presence of a rigid lamina cribrosa, can induce a compression of the capillaries with a secondary ischemia of the ON axons, in the context of a compartment syndrome [3,5]. Using OCT imaging, previous authors found that a smaller C/D ratio is a poor prognostic marker in NA-AION patients [33]. The absence of a crowded OD in the fellow eye at the onset should warrant an investigation for an alternative diagnosis, especially A-AION, for which a crowded OD is not a prerequisite [46,47];
- ***Optic disc drusen***: OD drusen consists of calcificated aggregates of extracellular materials accumulating as a consequence of axoplasmic transport alterations at the ONH and can be easily demonstrated using OCT and fundus autofluorescence imaging. OD drusen is usually asymptomatic and diagnosed as an incidental fundus finding. It can be associated with transient visual obscuration, likely due to temporary impairment of the ONH circulation or with slowly progressive VF loss, especially an enlarged blind spot, arcuate defects, and peripheral depression, that are thought to be caused by direct axonal compression. In rare cases, OD drusen have been associated with central retinal artery and vein occlusion and with NA-AION, especially in patients younger than 50 years [15,35,37]. A recent multicenter retrospective study showed that, in NA-AION patients younger than 50 years, OD drusen identified with OCT imaging were present in more than 50% of cases [35]. It is supposed that the OD drusen may cause an NA-AION by inducing an exaggerated OD crowding, but it remains unclear as to why this is a relatively rare occurrence;
- ***Optic disc edema due to any cause:*** other types of OD edema have been associated with NA-AION, for example, in cases of papilledema induced by raised intracranial pressure [48];
- ***Vascular abnormalities of the ONH feeding vessels:*** have been linked to NA-AION development in rare cases [7,12];
- ***Acute and subacute IOP increase***: previous case reports have associated NA-AION with the acute IOP increase caused by acute angle closure glaucoma, likely due to an OPP decrease below the critical range of autoregulation functioning [49]. Moreover, it is supposed that an IOP increase may induce an OPP reduction in the absence of efficient vascular autoregulation mechanisms [18], with consequent impairment of the ONH blood supply [23]. For example, the well-known IOP increase during the supine or lateral decubitus during sleep, likely due to the increase of the episcleral venous pressure, is supposed to significantly reduce the OPP of the ONH, especially in the presence of exaggerated nocturnal arterial hypotension, “disk-at-risk” or impaired blood-flow autoregulation, predisposing some susceptible subjects to an ONH ischemic insult [50]. Measuring IOP and systemic blood pressure in patients with unilateral NA-AION, Yang et al. found that there was a significant increase in IOP and decrease in OPP after changing position from a supine to lateral decubitus position at the affected eye site, suggesting that the posture-induced IOP may be a risk factor for NA-AION development [51]. Supporting this hypothesis, it should be noted that several cases of NA-AION manifest upon awakening [52]; therefore, a link with sleeping body posture could be plausible;
- ***Acute arterial hypotension and acute hypovolemic episodes*:** NA-AION is described as a rare complication of acute bleeding, shock events, and hemodialysis [53,54]. An acute systemic hypotension that causes an abrupt reduction in the OPP beyond the critical range of autoregulation mechanisms, or, in the absence of efficient autoregulatory mechanisms [18], may induce a significant ONH blood supply reduction [23];
- ***Nocturnal systemic arterial hypotension***: considering that the acute vision loss at NA-AION presentation is noticed upon awakening in more than 70% of cases [52], it is suggested that nocturnal hypotension could be a precipitating risk factor for NA-AION, especially in so-called “deeper” subjects, i.e., subjects in which the physiological nocturnal hypotension occurring during sleep, due to the attenuation of the sympathetic tone, is significantly higher than in normal subjects; or in patients assuming anti-hypertensive medications at night. The effective role of the nocturnal systemic hypotension remains unclear and controversial. Comparing the 24 h blood pressure data amongst NA-AION, POAG and NTG patients, Hayreh et al. suggested that nocturnal systemic hypotension may have a role in the development of NA-AION in susceptible subjects [55]. On the other hand, Landau et al. [56] compared the 24 h blood pressure in NA-AION and controls matched for age, associated disease, and medications, and found a similar nocturnal decrease in blood pressure in the two groups, but a slower morning rise in pressure in NA-AION patients, that could explain the typical presentation of NA-AION upon awakening;
- ***Metabolic syndrome***: metabolic syndrome is a clinical entity including three or more of the following clinical features: systemic hypertension, diabetes mellitus, hypertriglyceridemia, hypercholesterolemia and central adiposity [57]. Metabolic syndrome and its components may cause vasculature alteration (atherosclerosis and arteriosclerosis), blood-flow impairment, and autoregulatory dysfunction, and are risk factors for cerebral and cardiovascular disease and increased mortality [57]. Metabolic syndrome has been found to increase the risk of NA-AION by twofold [57]. NA-AION has been demonstrated to be significantly associated with systemic arterial hypertension, found in 35–50% of patients [14,58]; diabetes mellitus, present in 5–25% of cases [14,34,58,59]; hyperlipidemia, hypercholesterolemia and hypertriglyceridemia, atherosclerosis, and arteriosclerosis [60]. Undetected or untreated systemic hypertension and diabetes mellitus are the most important underlying disease amongst NA-AION patients (cardio);
- ***Cardiovascular and cerebrovascular diseases***: in comparison with a matched population, patients developing NA-AION have been demonstrated to be at higher risk of acute cerebrovascular and cardiovascular events, such as stroke, transient ischemic events, hearts attacks, and are also at a higher risk of dying from vascular events [14,58,61,62]. A recent retrospective study found that patients with NA-AION have a 3.35 times increased risk of developing an ischemic stroke when compared with patients with similar comorbidities but without NA-AION [61]. The association between NA-AION and cardiovascular and cerebrovascular diseases strongly suggests that ocular signs and symptoms of ocular arterial ischemic diseases may be a warning sign of stroke or heart attacks;
- ***Carotid stenosis***: an association with NA-AION has been demonstrated by previous authors [63] and rejected by others [64]. It is not clear if the internal carotid artery siphon region narrowing frequently found in NA-AION patients could contribute to the pathogenesis of the NA-AION or could be a result of NA-AION disease because of the reduced blood-flow demand by the atrophic tissue, rather than the cause [63];
- ***Chronic renal failure and dialysis***: a relationship between end-stage renal disease and NA-AION has been widely reported [65] and may have the following explanations: the chronic hypertension typically affecting these patients may impair the autoregulation of the ONH blood flow; the secondary chronic hypotension and anemia found in patients that have undergone several hemodialytic treatments may reduce the ONH perfusion pressure and oxygenation, causing the so-called “dialysis-associated NA-AION” [65]. In cases associated with intradialytic acute severe hypotensive episodes, bilateral simultaneous NA-AION involvement has been reported in approximately 25% of cases [65];
- ***Migraine:*** a statistically significant association has been found between NA-AION and migraine, in particular in patients younger than 50 years [15,66]. Visual loss, typically occurring during or immediately after the episode of cephalgia, is supposed to be related to a vasospasm of the ONH vessels. Previous authors have suggested that the beta-blocking agents used to treat migraine may potentiate this vasospastic effect by disrupting the vasoregulatory mechanisms [66];
- ***Smoking***: the relationship between smoking and NA-AION is controversial. Although previous studies did not find any relationship [7], Chung et al. [67] found that smokers developed NA-AION at an age significantly younger than nonsmokers;
- ***Obstructive sleep apnea syndrome***: the obstructive sleep apnea syndrome (OSAS) is a sleep disorder with nocturnal pharyngeal collapse inducing a partial airway obstruction with hypopneic or apneic events during sleep. The hypoxia, hypercapnia and acute blood pressure spikes induced by the nightly transitory cessation of breathing present in OSAS patients have been demonstrated to increase the risk for coronary artery disease, heart failure, stroke, and ocular manifestations. In particular, a significant link between OSAS and NA-AION has been widely reported in the literature [68,69]. Previous studies have shown that OSAS is present in up to 89% of NA-AION patients [68,69]. Moreover, recent large studies have reported that the risk of developing NA-AION was increased by 1.7–3.8-fold in OSAS patients as compared to controls [68]. Finally, OSAS seems to be a risk factor for second eye involvement in NA-AION [68]. The pathogenesis of NA-AION in the presence of OSAS is still debated. Three concomitant mechanisms have been advocated: a transient hypoxia, an impaired blood flow autoregulation, and an increase in intracranial pressure during the apneic episodes, with subsequent reduction in the OPP at the ONH level [69]. At any rate, it is still unclear if the treatment of OSAS with the C-PAP can reduce the risk of first or fellow eye involvement [69,70];
- ***Hypercoagulative states and congenital or acquired thrombophilia***: although the relationship between NA-AION development and thrombotic risk factors remains unclear, several data suggest that some previously undetected pro-thrombotic conditions could be linked to the NA-AION onset. The risk of ischemic events [14]. The hyperhomocysteinemia has been associated to ischemic events (stroke, myocardial infarction, peripheral vascular disease) in patients younger than 50 years. The link between NA-AION and hyperhomocysteinemia has been widely investigated but remains unclear [14,71]. As compared with a control group, NA-AION patients showed a higher platelet count and higher mean platelet volume, indicating a greater platelet activation and suggesting a pre-thrombotic or hypercoagulative status in NA-AION patients [39,72]. Platelet glycoproteins polymorphisms, especially the polymorphism of the GPIba gene, which encodes for a subunit having a fundamental role in the interaction between platelet and vascular endothelium during the thrombus formation, have been associated with higher risk of first and second eye involvement in NA-AION [73]. On the other hand, the relationship between NA-AION and other risk factors for thrombosis, including lupus anticoagulants, anticardiolipin antibodies, prothrombotic polymorphism (factor V Leiden, angiotensin-converting enzyme, and angiotensin II receptor polymorphisms), deficiencies in protein C and S and antithrombin III, although documented by isolated reports, were not confirmed by large-scale studies [12,14];
- ***Vitreopapillary traction*:** previous authors have suggested that vitreopapillary traction (VPT) and total or partial posterior vitreous detachment (PVD) could be associated with the development of NA-AION [74], so that the term of “papillary vitreous detachment neuropathy” has been proposed [74]. The underlying mechanism is supposed to be a partial or total PVD with abrupt VPT causing an axonal dynamic stretch injury with axonal cytoskeletal and membrane fracture and blockage of the axoplasmic flow [74]. The onset of an asymptomatic and reversible OD edema or the comparison of signs and symptoms of neuropathy is thought to be dependent on the axonal injury severity [74]. This cascade of events is suggested to be more likely in older age groups, when the axons became less elastic; in “disk-at-risk”, having firmer vitreoaxonal attachments; and in diabetic patients, where a precocious vitreous syneresis may precipitate earlier PVD and explain the so-called diabetic papillopathy [74]. In the presence of a partial PDV and “papillary vitreous detachment neuropathy”, some authors have performed a via pars plana vitrectomy to cut the VPT and reported promising results in terms of increased VA in treated cases [75]. This issue is still debated, and, at present, clinical and instrumental (OCT) data are insufficient to demonstrate the causal role of VPT in the development of NA-AION [76]. In particular, it has been underlined that DPV and NA-AION have significantly different risk factors, visual symptoms related to PVD (myodesopsias and phosphenes) are not typically reported before NA-AION presentation, and no cases of OCT-documented VPT at the NA-AION onset have been reported; moreover, a pre-existent PVD, before the NA-AION onset, has been frequently documented [76]. VPT-induced optic neuropathy, characterized by segmental OD swelling and surface vessel telangiectasia, has been previously described in the literature and could represent a separate clinical entity that may be confused with the “incipient NA-AION” form [76].
- ***Uncomplicated ocular or non-ocular surgery (peri-operative or post-operative or surgical NA-AION):***
  ***a.***  ***perioperative NA-AION associated with ocular surgery***: several case reports of NA-AION presenting hours, days, or months (with a median latency of 6–12 weeks) after an uncomplicated cataract extraction, the so-called “post-cataract extraction NA-AION”, have been described [77,78]. Lam et al. [77] calculated that patients developing a “post-cataract extraction NA-AION” have a 3.6 times higher risk of experiencing the same form in the fellow eye after the same procedure. A recent review and meta-analysis found that the risk of NA-AION after cataract surgery is four times greater within the first year post-operatively and usually occurs within six months, with an incidence of <1 for 1000–3000 surgeries [78]. Although the pathogenesis of these cases is yet unclear, an acute intraoperative IOP spike abruptly reducing the OPP of the ONH in predisposing subjects seems to be the more likely mechanism. Moreover, a significant association with retrobulbar anesthesia administration has been noted; it has been postulated that the delayed hematoma expansion caused by the retrobulbar injection may play a role [77]. Delayed forms of perioperative NA-AION, occurring months after surgery, are supposed to be related to postoperative inflammation, and this hypothesis is supported by the evidence that these cases had a higher frequency of surgical complications, and are linked to prolonged surgical times and greater inflammation [78]. Finally, NA-AION cases have been described after uncomplicated intravitreal injection of anti-vascular endothelial growth factor (VEGF) agents [79]. In these cases, beyond the acute short-term increase in IOP following the intravitreal injections, the vasoconstrictor effect of the anti-VEGF may also be advocated [79];
  ***b.***  ***The perioperative NA-AION associated with non-ophthalmic surgery:*** is most commonly a PION (>50% of cases), and less often an AION [80]. The most common surgical procedures associated with ION are cardiac surgery and spinal fusion [80]. Cardiac bypass surgery is associated with a risk of NA-AION in up to 1% of cases [80]. The symptoms, typically complained 1–2 days after surgery, are frequently bilateral (over than 60% of cases) and severe, with VA reduced to light perception in more than 50% of cases [80]. Risk factors associated with perioperative ION in spinal and cardiac surgery are male gender, obesity, long anesthesia duration, blood loss, transfusions, prone position, peripheral vascular disease, anemia, diabetes, and stroke [80];
- ***Drugs:*** several drugs have been associated with the development of NA-AION, which include:
  ***a.***  ***phosphodiesterase type-5 inhibitors (PDE-5i):*** sildenafil, tadalafil and vardenafil (Viagra, Cialis, and Levitra), widely prescribed for erectile dysfunction and pulmonary hypertension, have been associated with NA-AION occurrence [81,82]. Although the causal relationship is still controversial [82], these substances are thought to increase the risk of NAION by causing vasodilation and lowering the perfusion pressure to the ONH in the presence of structurally predisposed OD. Indeed, all reported cases showed a crowded OD [82];
  ***b.***  ***interferon-alpha:*** several studies have reported a temporal association between therapy with interferon-alpha and the development of a usually bilateral NA-AION [83]. Suggested pathogenetic mechanisms include the systemic hypotension caused by the interferon or an induced immune complex deposition within the OD circulation [83];
  ***c.***  ***amiodarone:*** several authors have described an association between NA-AION and the assumption of amiodarone [84]. NA-AION related to the treatment with amiodarone are mostly described as bilateral, with an insidious onset, generalized rather than altitudinal VF defect, and OD swelling persisting for months rather weeks after the onset of the visual loss [84]. It is important to note that patients treated with amiodarone generally have severe vascular risk factors that may predispose them to the development of NA-AION; a clear association with the therapy with amiodarone remains uncertain;
  ***d.***  ***oral contraceptives:*** the use of oral contraceptives has been associated with rare ocular complications, including NA-AION cases [85];
  ***e.***  ***sumatriptan***: the use of sumatriptan for migraine has been linked with the development of NA-AION, and is likely caused by the vasoconstrictory effect of the drug [86];
  ***f.***  ***drugs used to treat cardiovascular diseases:*** the use of anti-thrombotic agents, beta-blockers, statins, aggressive anti-hypertensive therapy and night-time dosing of anti-hypertensives have been linked with the development of NA-AION [14]. The causal association is obviously unclear considering that these drugs are used in subjects with vascular risk factors that may act as predisposing or precipitating factors for NA-AION;
- ***Infections and vaccinations:*** NA-AION cases have been described soon after various infections, including syphilis, rickettsia, hepatitis C virus, borrelia burgdorferi, herpes simplex, Chlamydia pneumoniae [87,88] and severe acute respiratory syndrome coronavirus 2 (SARS-CoV-2] infection, the causal agent of COVID-19 disease [89,90]. Moreover, an association between NA-AION and vaccinations against influenza [91] and COVID-19 disease has been reported [92,93]. The majority of the published cases are linked to the SARS-CoV-2 virus infection and to the vaccines against it [89,90,92,93]. Although it remains uncertain whether the relationship between NA-AION and COVID-19 infection or vaccination may be consequential or coincidental, possible causative mechanisms include: inflammatory or autoimmune thrombotic microangiopathy and endothelitis; hypercoagulability due to platelet activation; and severe hyper- or hypotension episodes likely due to a dysregulation of the renin–angiotensin system [89,90,92,93,94];
- ***Genetic factors*:** the role of hereditary factors in the development of NA-AION remains largely unknown. The data supporting a link with genetic factors include: platelet glycoprotein polymorphisms, especially polymorphism of the GPIba gene, have been associated with a higher risk of first and second eye involvement in NA-AION [73]; rare familial cases of NA-AION have been described, some of theme associated with mitochondrial mutation GA132A, which are characterized by an earlier onset and a higher frequency of bilateral cases [95]; factor V Leiden mutation, inherited following an autosomal dominant trait, is one of the most common causes of inherited thrombophilia and has been associated with the development of NA-AION [96]; specific haplotypes and gene polymorphisms, including ACE I/D and MTHFR 6ìC677T, have been linked to a higher risk of developing NAION, indicating that this disorder may have a hereditary predisposition [97];
- ***Autoimmune and inflammatory diseases***: NAION may also be related to some autoimmune and inflammatory diseases such as giant cell arteritis, lupus, and sarcoidosis [98]. The pathogenetic mechanism is presumed to be inflammation and damage to the ONH feeding vessels;
- ***Miscellanea:*** other factors have been linked to the NA-AION development, including anemia; chronic obstructive pulmonary disease; hypothyroidism; age-related macular degeneration; and glaucoma [14].

## 6. Clinical Signs and Symptoms

The distinctive clinical presentation of the NA-AION is an acute unilateral painless vision loss (i.e., visual field defect ± visual acuity loss) associated with a sectorial or diffuse ONH edema [1,2,3,4,5,6,11,45].

NA-AION clinical signs and symptoms include:

- ***Visual acuity (VA) loss:*** visual acuity is typically:
  ***(a)***  ***acute***
  ***(b)***  ***unilateral:*** VA loss in NA-AION is typically unilateral. Fellow-eye involvement can be contemporary or sequential. The bilateral simultaneous form is rare and typically associated with acute severe systemic hypotension, extraocular surgery, or drugs assumption [53,65,80,83]. The sequential involvement of the fellow eye has been reported to occur in 15–30% of cases within five years of the first eye damage, with a median time between first and second eye involvement of 7–12 months [99]. The sequential occurrence of NA-AION in the contralateral eye, with abrupt vision loss and OD edema in one eye and OD atrophy in the contralateral one, can configure the clinical appearance of the so-called “pseudo-Foster Kennedy syndrome”; conversely, the true Foster Kennedy syndrome is characterized by a pale OD from a compressive etiology in one eye and an edematous OD secondary to papilledema from increased intracranial pressure in the fellow eye, without any history of abrupt vision loss;
  ***(c)***  ***variable in severity***: VA at the onset can vary from 10/10 to perception of light, being 10/10 in 20–33% of cases, better than 5/10 in more than 50% of cases and ≤1/10 in approximately 20–33% of cases [34,100]. Very poor VA (hand motion or worse) at presentation is unusual in NA-AION, found in 3.5–14% of cases [101], and should raise the suspicion for A-AION [46,47];
  ***(d)***  ***noted upon awakening*** in more than 70% of cases [52];
  ***(e)***  ***stable*** in approximately 2/3 of patients; in the remaining 1/3 of cases, VA loss can show a steady or episodic rapid progressive decrease over days to 6–12 weeks before a stabilization [100]. In cases where the visual loss is progressive, the patients frequently noticed the vision deterioration upon awakening in the morning [11]. The progressive form is thought to be due to the compartment syndrome at the ONH level, with compression of the feeding vessels and secondary ischemia of ONH [7,8,9,17];
- ***Unilateral acute stable or rapidly progressive visual field (VF) defect*:** is always present upon presentation and it is described as blurring in the affected VF region [11]. Although any pattern related to the ONH damage can be present, altitudinal loss, usually inferior, and the arcuate defect occur in 55–80% of cases; moreover, 20–25% of cases show central scotomas [34,102]. The most common VF defect pattern found in NA-AION seems to be a combination of a relative inferior altitudinal defect with an absolute inferior nasal defect [103]. The frequency of altitudinal VF defects in NA-AION supports the proposed semicircle organization of the SPC arteries feeding the ONH [19]. Differently from VA loss, which can be absent in 1/3 of cases, the VF defect is always present at the onset of the disease, so perimetry represents the most important diagnostic test in NA-AION [11,103];
- ***OD swelling***: is always present at onset, being part of the diagnostic definition of NA-AION [2,4,7]. It may be diffuse or segmental, hyperemic, or pale; sometimes a sharply demarcated horizontal linear border is present [104]. The onset of the OD edema is assumed to be simultaneous to the vision loss. Several previous authors have described the so-called “incipient NA-AION”: it is an asymptomatic OD edema that can spontaneously resolve, which is explained as reversible ONH ischemia without infarction, and, in 25–45% of cases, can progress to an overt NA-AION within a few weeks [32]. This clinical entity has been also described in the fellow eye of NA-AION patients [105]. A pre-symptomatic phase of NA-AION with impaired perfusion of the ONH has been confirmed with fluorescein angiography [15]. The OD edema, characterized by the acute NA-AION phase, typically resolves within 6–11 weeks. While the OD edema is present, VA can continue to decrease because of the compartment syndrome effect [7,8,9,17]. The OD swelling is replaced by a sectorial or diffuse OD pallor due to atrophy, that starts to develop 2–3 weeks after the onset of the visual loss [104]. OD edema persisting over 11 weeks should suggest an alternative diagnosis, such as compressive or infiltrative optic neuropathies.

In the end stage of AION, an excavation of the ONH, called cavernous degeneration of Schnabel, can develop. This form, clinically indistinguishable from that seen in glaucomatous optic neuropathy (GON), is relatively uncommon in the NA-AION form (2% of cases) and typically presents in the chronic stages of A-AION (92% of cases) [106].

- ***Relative afferent pupillary defect (RAPD)***: this is commonly present in unilateral cases, and may be present and asymmetric in bilateral cases;
- ***Absence of pain*:** an ocular discomfort unrelated to eye movements is reported in 8–12% of cases. The coexistence of OD edema and pain is atypical in NA-AION and should induce the suspicion of other diagnoses, including GCA, where patients frequently complain of headaches; idiopathic and demyelinating optic neuritis (multiple sclerosis and neuromyelitis optica), in which patients commonly complain ocular pain that worsens with eye movements; immune-mediated optic neuritis (sarcoidosis, Wegener’s granulomatosis); and neoplastic optic neuritis (myeloma, germinoma, fibrous dysplasia; infective optic neuritis (bacterial, viral, TBC) [107];
- ***Crowded OD in the fellow eye***: a small OD in diameter with a small or absent physiologic cup is observed in 80–90% of cases. A “crowded” OD is considered to be an important risk factor for the development of NA-AION [14,34], especially in patients younger than 50 years [15];
- ***Peripapillary retinal hemorrhages***: these are very common (3/4 of cases), especially in diabetic patients [15];
- ***Color vision loss:*** impaired color perception is a sensitive sign of ON dysfunction and, in comparison with other ocular diseases, in optic neuropathies, the color vision is affected to a more significant degree at any level of VA. In NA-AION, it is typically proportional to the VA loss; in contrast, patients affected by optic neuritis characteristically show a color vision loss greater than the VA loss [108];
- ***Photophobia***: is a common complaint, especially in bilateral cases [11];
- ***Diffuse or segmental narrow of the retinal arterioles***: is a common feature, especially in more severe cases;
- ***Macular edema and submacular fluid accumulation*** may also occur;
- ***OD surface vascular dilatation*** can be present occasionally, especially in diabetic patients [15];
- ***Cotton wool spots*** are uncommon;
- ***Hard exudates forming a macular star*** are uncommon and should suggest other clinical conditions, such as neuro-retinitis;
- ***simultaneous occlusion of the cilioretinal artery*** is exceptional [109], being a typical hallmark of A-AION [46,47].

NA-AION patients younger than 50 years are not rare, representing 7.5–25% of NA-AION cases [15], and are reported more frequently amongst Asian populations [13]. The so-called “NA-AION of the young” seems to have some peculiarities [15], including:

1. ***higher association with***: crowded OD, present in approximately 88% of cases and is the only risk factor present in 25% of cases; renal failure and dialysis; migraine; diabetes; systemic hypertension; and a hypercoagulable state;
2. ***higher rate of ipsilateral eye recurrence***;
3. ***more frequent fellow eye involvement***, occurring in approximately 40% of cases. The risk factors associated with fellow eye involvement in patients younger than 50 years were diabetes, anemia, and chronic renal failure;
4. ***more frequently found fluorescein angiographic features of OD ischemia.***

## 7. Diagnosis and Differential Diagnosis

### 7.1. Diagnosis

NA-AION diagnosis is primarily clinical, and is commonly based on the following clinical features: acute monocular painless visual loss; presence of RAPD; sectorial or diffuse OD edema; peripapillary hemorrhage; and arciform or altitudinal VF defects. The patient’s medical history and the exclusion of other possible etiologies are of crucial importance [1,2,3,4,5,6,45,110].

Typical clinical features at the onset or during the clinical course that make the diagnosis of NA-AION highly likely, and atypical features that suggest investigating and excluding diagnoses other than NA-AION, are summarized in Table 1.

### 7.2. Differential Diagnosis (DD) in the Acute NA-AION Stage

In the acute NA-AION phase, all other causes of unilateral or bilateral (less frequent) OD swelling need to be investigated and ruled out [111], especially if atypical clinical features are present (Table 1).

The following clinical entities should be especially excluded (Table 2):

***a. A-AION*:** in the presence of a possible diagnosis of AION in patients older than 50 years, the first fundamental step is to exclude an A-AION. A-AION is almost always due to giant cell arteritis (GCA) [11,110], which represents a vision- and life-threatening condition and one medical emergency in ophthalmology: its prompt diagnosis and treatment with high-dose steroid therapy may indeed prevent further vision loss in the interested eye and reduce the risk of fellow-eye involvement and severe systemic complications [46,47].

GCA is an autoimmune-mediated process with several features suggesting a connection with an infectious agent, likely the varicella zoster virus [46,47]. It causes a T cell-mediated granulomatous systemic vasculitis that mainly involves medium-sized and large arteries and, in the eye, shows a special predilection to involve the SPC arteries (20% of cases) [47], inducing SPC arteries thrombotic occlusion and ONH ischemia that have widely histopathological demonstrations [16].

A-AION is significantly less frequent than NA-AION, with an estimated annual incidence of 0.36 per 100,000 population in patients older than 50 years [1,11,46,47]. The clinical presentation of A-AION is similar to that of NA-AION, with acute unilateral VA loss and OD edema. At any rate, a younger patient age, male sex, better initial VA, the presence of at-risk OD in the fellow eye, and better spontaneous visual recovery generally propend towards the diagnosis of NA-AION, whereas advanced age, female sex, poor VA at onset frequently preceded by amaurosis fugax or diplopia, the presence of pain and systemic symptoms, the presence of systemic signs of flogosis, FAG features of OD, and choroidal ischemia suggest the diagnosis of A-AION [1,11,46,47] (Table 1 and Table 2). An acute VA loss with RAPD and without OD edema suggests PION. In this case, it is crucial to exclude a GCA, because non-arteritic PION is very rare [5].

The diagnostic features for A-AION include [1,11,46,47]:

1. Chalky white OD edema
2. SPC arteries occlusion as demonstrated with FAG: in A-AION, a severe, diffuse filling delay of both OD and peripapillary choroid can be demonstrated, suggesting a blood-flow impairment at the level of the SPC arteries before the bifurcation into parapapillary and choroidal branches;
3. The presence of associated cilioretinal artery occlusion;
4. Abnormally high pre-treatment erythrocyte sedimentation rate (ESR) and C-reactive protein (CRP) levels, which are a diagnostic hallmark of GCA and have shown a sensitivity of, respectively, 86% and 97.5% in identifying biopsy-positive GCA and discriminating between A-AION and other optic neuropathies, even if the so-called “inflammatory marker negative” GCA rarely occurs [112].

The temporal artery biopsy is the gold standard for the diagnosis of A-AION and should be performed in all suspected cases. High doses of steroids, orally or intravenously administered, are mandatory in A-AION and should be started immediately once the diagnosis is suspected and tapered very slowly, guided by symptoms, CRP, and ESR, over the course of months or years [113]. Immunosuppressive therapy should be considered in the case of steroid-related side effects [113]. A-AION prognosis is commonly severe, only 4% of patients will improve their visual function with steroids, and 4% deteriorate visually even with steroids [1,11,46,47]. Fellow-eye involvement occurs in approximately 50% of untreated cases within a few days or weeks [1,11,46,47];

***b. Papillitis or anterior or intraocular optic neuritis***: is an acute inflammation of the ON that involves the ONH, characterized by unilateral acute VA loss and VF defects, RAPD, OD edema, and pain. It can be classified into different forms, including: idiopathic; demyelinating (multiple sclerosis and neuromyelitis optica); infective (bacterial, viral, TBC); immunomodulated (sarcoidosis, Wegener’s granulomatosis); and neoplastic infiltrative (myeloma, leukemia, lymphoma, germinoma, fibrous dysplasia) [107,114]. The most common papillitis are idiopathic, demyelinating, and antibody-mediated (aquaporin-4 and antimyelin oligodendrocyte glycoprotein (MOG)). Patients with idiopathic optic neuritis have a 50% chance of developing multiple sclerosis within 15 years of disease presentation [107,115].

NA-AION and optic neuritis are the most common acute optic neuropathies in adults and the most common causes of unilateral OD edema [111]; most importantly, they may have an overlapping clinical profile that can sometimes make clinical differentiation a challenge [114,116,117]. However, younger age, female sex, better initial acuity, hyperemic OD edema, central scotoma, pain with eye movement, no FAG feature of OD ischemia, early recovery of VA and good response to treatment with intravenous steroids with an overall better long-term prognosis generally propend for the diagnosis of optic neuritis; whereas advanced age, male sex, poor initial acuity, altitudinal VF defect, segmental OD edema, absence of pain, presence of FAG signs of OD and/or peripapillary choroid ischemia, less favorable long-term prognosis with less improvement in VA, worse response to steroids and the presence of systemic hypertension or diabetes suggest the diagnosis of NA-AION [114,116] (Table 1 and Table 2). Rizzo & Lessell reported that, amongst their NA-AION patients, 8% complained of pain 26% had a central scotoma, whereas 10% of the optic neuritis patients had an altitudinal VF defect [116]. Patients with MOG-associated optic neuritis have a typical OD swelling with hemorrhages upon onset but, differently from NA-AION patients, they show a rapid recovery of visual function in the majority of cases, with a good final prognosis [116,117];

***c. Diabetic papillopathy*:** the currently accepted criteria for the diagnosis of this clinical entity include: the presence of diabetes type 1 or 2; unilateral or bilateral OD edema, frequently hyperemic and with marked dilatation of the OD surface microvasculature; the absence of OD dysfunction, except for a minor RAPD; minimal VF defects with no altitudinal pattern; and lack of evidence of ocular inflammation or elevated intracranial pressure [15,118]. A crowded OD in the fellow eye is frequently present [15]. The OD edema may persist for up to 12 months, after which it resolves spontaneously, leaving little or no OD atrophy [15,118]. FAG features of ischemia are frequently evident [15]. The pathogenesis of this form is unclear, and several authors have postulated a mild and reversible form of OD ischemia of the prelaminar ONH region [15]. Previous authors have suggested that OD ischemia may have a wide spectrum, ranging from OD edema without nerve dysfunction to overt OD infarction (NA-AION) with permanent VF loss [15]. Both “incipient NA-AION” and diabetic papillopathy demonstrated FAG features of OD ischemia and could be considered IONs with minimal and reversible OD ischemia, without ONH axon infarction [11,15,118];

***d. Compressive optic neuropathy***: several anterior orbital lesions, including tumors, may induce ON compression with consequent swollen OD and visual loss [111]. The typical clinical features in these cases are a gradual and progressive visual loss associated with other signs of orbital disease, including mild proptosis, lid or eye movement abnormalities and other neurological symptoms, pain that worsens with eye movement, and persistence of OD edema after 4–6 weeks, which is unusual in NA-AION. No FA features of OD ischemia are present (Table 2);

***e. ONH drusen*:** frequently bilateral and asymmetric, may mimic OD edema, but the visual function is generally normal. It may be associated with slowly progressive VF loss, likely due to ON axon compression. It has been linked to the development of NA-AION in rare cases, especially in patients younger than 50 years [15,35,36];

***f. Bilateral OD edema:*** these forms are rarely included in DD with NA-AION because the bilateral simultaneous forms of NA-AION are infrequent and typically associated with severe systemic hypotension, extraocular surgery, and the use of drugs [53,65,80,83]. Patients with bilateral OD edema and normal visual function most likely have papilledema or systemic severe hypertension [111]. Papilledema, i.e., the OD edema secondary to elevated intracranial pressure, is typically associated with symptoms of high intracranial pressure. The DD requires neuroimaging and lumbar puncture. The papilledema related to systemic hypertension is associated with signs of hypertensive retinopathy. Patients with bilateral OD edema and abnormal visual function most likely have bilateral demyelinating optic neuritis, neuromyelitis optica spectrum disorder, and MOG, and require investigation with contrast-enhanced MRI of the brain and orbits [111].

### 7.3. Differential Diagnosis in the Chronic NA-AION Stage

In this phase, the DD may include all other causes of ON atrophy. Moreover, considering that an OD cupping could be rarely present, glaucomatous optic neuropathy (GON) and the other causes of non-glaucomatous OD excavation should be ruled out.

***a. OD atrophy:*** appears as a pale OD on fundus examination and represents the hallmark of the damage of the anterior visual pathway [119]. The causes of OD atrophy include congenital optic neuropathies; extrinsic ON compression; intrinsic ON tumors; vascular disease, including AION, central retinal artery occlusion, carotid artery occlusion; inflammatory, infective, toxic, traumatic optic neuritis, and papilledema. The DD can be difficult and requires investigation with contrast-enhanced MRI of the brain and the orbits [119];

***b. Glaucomatous optic neuropathy (GON):*** NA-AION, in its post-acute phase, is the non-glaucomatous optic neuropathy more frequently confused with GON, especially when the clinical history is unclear and the IOP is within the normal limits, i.e., in the presence of a normal tension glaucoma [120]. The evolution toward an OD cupping, frequently seen in A-AION (more than 90% of cases) and rare in NA-AION eyes (2% of cases) [106], can be associated with altitudinal or arciform VF defects, making the DD from GON a challenge [120]. The DD can be difficult and may require a long follow-up, considering that untreated GON is, by definition, a progressive form.

### 7.4. What Kind of Tests May Be Useful for the Differential Diagnosis?

***a. blood exams***: should include a full blood count, erythrocytes sedimentation rate (ESR), C-reactive protein (CRP), platelet count, and homocysteine levels in patients under 50 years of age. As is known, high levels of ESR and CRP are diagnostic for GCA [1,11,46,47];

***b. Fluorescein angiography (FAG):*** although no typical FAG features of NA-AION have been identified (A10, A100 and A77), a pattern consistent with OD ischemia may be found in 70–80% of NA-AION cases, ranging from mild perfusion delay to severe impaired perfusion [11,15]. The FAG features of OD ischemia in NA-AION consist of a characteristic OD early filling delay (55–75% of cases), an OD early segmental hyperfluorescence (50–70% of cases) and a circular or localized or watershed zone pattern peripapillary choroidal filling delay during the very early choroidal arterial phase (25% of cases); a late leakage from the OD is also frequently found [11,15,27] (36 di A102). Mild FAG features of OD ischemia are present in the “incipient NA-AION” and diabetic papillopathy [15] as well, with the second entity showing also a typical very early leakage from the surface dilated vessels [15]. On the contrary, FAG signs of OD and peripapillary choroidal ischemia are typically absent in non-ischemic OD edema [28], for example, in optic neuritis. Furthermore, FAG images frequently show vasodilatation in the region of the OD spared from ischemia [11,15,27], supporting the hypothesis of the so-called “luxury perfusion” as an autoregulatory mechanism to increase OD perfusion in the presence of ischemia [17];

***c. Optical coherence tomography (OCT)*:** is a non-invasive exam that can offer extensive information about the architecture of the ONH. Even if diagnostic OCT features for NA-AION have not been yet recognized [3,45], OCT can be used to identify a disk-at-risk or OD drusen and to monitor the disease course, with the assessment of the OD edema or atrophy [37,41]. Previous authors have demonstrated that, in comparison with healthy eyes, those affected with NA-AION showed a significant increase in peripapillary RNFL thickness in the acute phase, due to the OD edema, and a significant reduction in the chronic phase due to the evolution towards optic atrophy [41]. Moreover, OCT in post-acute NA-AION has demonstrated that both RNFL and RGC loss correlate with VF loss [41]. Recent studies have shown that OCT may help in discriminating between post-acute NA-AION and GON, showing that the neuroretinal minimum rim width appears significantly thinner and the lamina cribrosa depth significantly greater in GON than in NA-AION eyes [121]. This difference increases with disease severity, suggesting distinct remodeling patterns in response to different insults, with a prevalence of glioarchitecture loss in GON, and glioarchitecture hypertrophy in NA-AION [122]. Moreover, a recent systematic review and meta-analysis of OCT features found that the superior RNFL thickness was significantly thinner in post-acute NA-AION eyes than in POAG eyes and that inferior RNFL thickness was significantly thinner in POAG than in NA-AION eyes [43]; these differences may suggest different pathophysiological mechanisms between the two diseases [43];

***d. OCT angiography (OCT-A):*** is a recent non-invasive technique depicting ocular vessel density. Using OCT-A, previous studies have demonstrated a significant segmental and global reduction in the peripapillary region and OD vessel density in eyes affected by NA-AION in comparison with healthy eyes, both in the acute and chronic stages [123]. Moreover, it has been demonstrated that, in the chronic stages, the vessel density of ONH and the peripapillary zone is directly related to the RNFL damage and VF loss [123]. Finally, a recent study has demonstrated that the temporal sector of the ONH showed the highest blood flow density in healthy subjects, and it seems to be the most damaged in NA-AION eyes [124], which could explain the predominance of nasal FV defects found in NA-AION [34,102,103];

***e. Magnetic resonance imaging (MRI) of the orbits and the brain with contrast (gadolinium) and fat suppression***: is mandatory in the presence of atypical features for NA-AIO, to exclude other pathologies. Orbits and brain RMI imaging in NA-AION show a normal intra-orbital and intracranial ON, with no abnormal enhancement or diffusion restriction of the OD and ON frequently associated with signs of cerebrovascular alterations, especially in older patients [115]. A post-contrast enhancement of the OD in the absence of focal-restricted diffusion seems to be specific for NA-AION and may reflect the so-called “luxury perfusion” of non-ischemic zones or may reflect vascular endothelial damage with loss of the blood–brain barrier [115]. In the case of inflammatory, compressive, or infiltrative optic neuritis, RMI images show intra-orbital ON swelling and enhancement [115,125], whereas ON sheath and orbital fat enhancement are highly suggestive of vasculitis related to GCA [46,47]. Moreover, MRI images of the brain and orbits are diagnostic for multiple sclerosis and other demyelinating diseases and in the presence of orbital lesions of various natures [125];

***f. Visual evoked cortical potential (VECP):*** VEP can identify anomalies in the visual pathway and can be useful in discriminating between NA-AION and optic neuritis. VECPs are known to be delayed in optic neuritis caused by the typical demyelination of the ON fibers. Comparing NA-AION and idiopathic or demyelinative ON patients, previous authors found that the wave P100 latency appeared significantly reduced in optic neuritis patients, whereas the P100 amplitude was significantly reduced in NA-AION patients [126];

***g. Eco Doppler of the carotid arteries:*** should be required, especially in the presence of ocular ischemic syndrome, retinal emboli, or neurological deficits;

***h. Computed tomography angiography (CTA):*** may be considered, especially in patients complaining of unilateral head or neck pain, to exclude a carotid dissection.

## 8. Prognosis

The severity of the vision loss and the degree of ON injury at presentation determine the prognosis of NA-AION.

Visual loss in NA-AION is typically acute and stable, although a further worsening in the first days after presentation is not uncommon; however, the clinical course of NA-AION stabilizes within a few weeks, at most in 2–3 months, in the majority of cases [100,127,128]. A progressive form has been described, in which the low point of vision loss is generally reached within 2–3 months [129]. Progressive visual loss after 2–3 months is extremely rare and should suggest the exclusion of other clinical entities, particularly compressive neuropathies.

The natural course of NA-AION cases has been investigated by previous large prospective studies. The IODNT study showed that NA-AION eyes were stable or had a spontaneous improvement of the VA without any surgical procedure [128]. In particular, in untreated NA-AION eyes having an initial VA of 20/64 or worse, during a two-year follow-up, the recovery of at least three Snellen acuity lines was observed in about 30% of cases; 20% had an additional vision loss of three or more lines of vision; and in 50% of cases, VA remained unchanged after onset [128]. On the other hand, VF damage seems to improve less commonly [130]. Hayreh et al., following the natural history of 386 NA-AION patients first seen within two weeks after onset and with a VA of 20/70 or worse, reported that, six months after clinical onset of the disease, VA and VF damage improved spontaneously in 40% and 25% of cases, respectively [100]. The final visual improvement was found to be worse in patients who were older or had multiple systemic vascular diseases, in particular diabetes mellitus and hypertension [131].

In general, NA-AION has a relatively bad prognosis: in the majority of cases, it results in permanent vision loss, with 40–50% of patients having significant visual impairment or being legally blind in the affected eye. The final VA of untreated NA-AION patients is highly variable, with 50% of patients reaching a VA of ≥20/30 and 25% of cases achieving a VA of ≤1/10 [100].

VA loss and the VF defects present in NA-AION have been demonstrated to significantly reduce the quality of life and work of Na-AION patients [132].

After visual stabilization, generally occurring within 2–3 months after the onset, the risk of recurrence in the same eye has been estimated to be 3–8% [129,133].

The involvement of the fellow eye is also a serious issue, considering that it has been reported to occur in 15–30% of cases within five years of the first eye damage, with a median time between episodes of 7–12 months [99]. Younger age, several initial VF damages, diabetes mellitus, chronic renal failure, and severe OSAS have been significantly associated with a higher risk of fellow-eye involvement [15,44]. Clinical findings of the first involved eye have been demonstrated to not be predictive for the prognosis of the second involved eye [1]. Moreover, asymptomatic fellow-eye involvement, with preserved VA and the presence of a VF defect compatible with NA-AION has been demonstrated in 10% of cases during a five-year follow-up [134].

## 9. Treatment and Prophylactic Options

Therapeutic and prophylactic approaches to NA-AON are still highly controversial [1,2,3,4,5,6,11,45,110].

Numerous surgical and medical therapeutic strategies have been attempted, which include:

### 9.1. Surgical Treatments

- ***Optic nerve head sheath decompression***: in 1989 Sergott et al. claimed that a surgical procedure called “optic nerve head sheath decompression” may improve VA in “progressive” NA-AION cases [135]. This surgical procedure involves the creation of two or more slits or a window in the tissue surrounding the ON, allowing the cerebrospinal fluid to escape to reduce the pressure surrounding the ON, disrupt the “compartment syndrome” effect, increase the vascular and axonal transport at the ONH level, and to save reversible damaged axons. This procedure gained favor worldwide not only in cases of progressive NA-AION but also in all types of NA-AION. The Ischemic Optic Neuropathy Decompression Trial (IONDT) was a prospective, controlled, randomized clinical trial proposed to assess if “optic nerve head sheath decompression” could be useful in NA-AION [34]. The study lasted two years, included 119 treated and 125 untreated NA-AION patients, and concluded that this surgical treatment was not effective and could be harmful [128], so the procedure was abandoned;
- ***Transvitreal optic neurotomy***: this surgical procedure includes a central posterior vitrectomy and a stab incision at the nasal margin of the OD to open the scleral canal, thus treating the compartment syndrome and reducing the compression of the ON axon feeding vessels. Promising results after this surgical procedure have been reported by a small, uncontrolled, nonrandomized study [136].

### 9.2. Medical Treatments

Several medical therapies have been used in NA-AION patients, which include:

- ***Corticosteroids (CS):*** because of their well-known anti-edemigenous and anti-inflammatory effects, the steroids have been proposed for the treatment of NA-AION to decrease capillary permeability and accelerate the OD edema resolution, thus reducing the compression of capillaries and axons, improving ONH blood flow and increasing the survival of ischemic axons. However, their use for the treatment of NA-AION is still controversial [137,138]. CS has been used for the treatment of NA-AION via systemic, periocular, or intraocular administration since the late 1960s, and the small uncontrolled studies reported a higher VA acuity improvement in patients treated with CS compared to the untreated ones [138].

In 2008, Hayreh & Zimmerman conducted a large prospective nonrandomized, “patient choice”, unmasked study in which 696 NA-AION patients, having VA of ≤20/70 and seen within two weeks from the onset, were selected to be treated with 80 mg/day of oral prednisone for 14 days followed by a two-month taper. At six months from the NA-AION onset, the authors observed a VA improvement in 70% of treated and 40% of untreated patients, and a VF improvement in 40% of treated versus 25% of untreated patients [137]. They concluded that treatment with systemic CS during the acute NA-AION phase was effective in improving visual function [137].

Several successive studies did not confirm the beneficial effects of CS on visual outcomes in NA-AION patients, especially in cases with very poor visual acuity at presentation [101].

A recent prospective randomized controlled double-blind clinical trial showed that the treatment with CS in acute NA-AION did significantly accelerate the resolution of the OD edema and improve the electrophysiological parameters of the ON, but did not improve the final visual outcomes and, conversely, could have harmful side effects such as depression, hyperglycemia, ocular hypertension, and pulmonary embolism [139].

Recent systematic reviews and meta-analyses found that the treatment with systemic CS did not significantly improve VA or VF in NA-AION patients [140].

Finally, the results of the intravitreal administration of CS (triamcinolone acetonide) for the treatment of NA-AION are still unclear [141];

- ***Aspirin****:* has been studied both as a primary treatment and as a preventive measure for recurrence in the same eye and for fellow-eye involvement in NA-AION patients. Large retrospective, case-controlled studies showed that the final VA and the rate of recurrence in the same eye were similar in NA-AION patients receiving aspirin before, during, and after the disease as compared with untreated patients [99,142].

There is some evidence that aspirin may reduce the risk of second eye involvement [143], but this finding is controversial and two large retrospective studies with long follow-up found no benefit from aspirin in reducing the risk of NA-AION in the contralateral eye [99,142].

Despite the lack of evidence of the benefits as a treatment or preventive measure, aspirin is still recommended by many physicians because of the vasculopathy risk factors associated with NA-AION [3,5,45];

- ***Intravitreal injection of anti-vascular endothelial growth factors (VEGF):*** the rationale for the use of the anti-VEGF agents is the attempt to reduce OD swelling and the secondary compression on the microvasculature and ON axons. The outcomes of the treatment of NA-AION with intravitreal injections of anti-VEGF are still debated [144]. Moreover, several cases of NA-AION have been described shortly after an intravitreal injection of an anti-VEGF agent for age-related macular degeneration [79], which could be related to the short-term rise of IOP induced by the intravitreal injections and to the vasoconstrictive effect of the injected substances, especially in patients with predisposing risk factors such as disk-at-risk or impaired vascular autoregulation;
- ***Anticoagulants and thrombolytics*:** multiple microembolization may play a role in the pathogenesis of NA-AION in rare cases [11]. Some beneficial effects of a therapy with heparin and warfarin in NA-AION patients have been demonstrated [145];
- ***Heparin-induced extracorporeal LDL/fibrinogen precipitation***: this procedure eliminates fibrinogen, LDL, cholesterol, and triglycerides from blood, decreasing plasma viscosity and improving microcirculation, and it has shown some efficacy in increasing the final VA in NA-AION patients [146];
- ***Neuroprotective substances***: neuroprotection is defined as a therapeutic strategy aiming to keep neurons alive and functional. Considering that RGC death is the final consequence of the ONH ischemia in NA-AION eyes, neuroprotection strategies have been suggested as a potential treatment [147,148,149].

Several substances, including granulocyte colony-stimulating factor, omega-3 polyunsaturated fatty acids, Rho-kinase inhibitors, mesenchymal stem cells, and astaxanthin have been demonstrated to reduce the RGCs apoptosis in NA-AION animal models [149]. Different molecules with supposed neuroprotective effects have been used in the treatment of NA-AION patients, but studies investigating their efficacy are limited by the small cohort of included participants or by the absence of a control group [147,148,149], and, although some encouraging results have been reached, further controlled clinical trials are required.

In particular:

***a.***  ***Levodopa/carbidopa***: are neurotransmitter and neuroprotective agents. Their use in NA-AION is controversial [150];
***b.***  ***Erythropoietin (EPO) solution by intravitreal injection***: EPO can reduce the RGC apoptosis in vitro and its intravitreal injection in a small cohort of NA-AION showed some improvement in VA [151];
***c.***  ***Brimonidine tartrate***: two small studies investigated the effect of the treatment with local brimonidine tartrate in NA-AION patients and did not find an improvement in visual function [152];
***d.***  ***Memantine***: seems to be able to improve VA in NA-AION patients as compared with the untreated group [148,149];
***e.***  ***Citicoline***: the administration of citicoline 500 mg/day of oral solution for six months was shown to be beneficial in preserving VA, VF, visual evoked potential, and RNFL thickness in AION patients [153];
***f.***  ***Captase 2 inhibitors*:** a clinical trial evaluating the use of intravitreal injections of QRK207, a captase 2 inhibitor preventing apoptosis, failed to show efficacy [148,149];
***g.***  ***Gum mastic extract RPh201***: subcutaneous injections of RPh201, an isolated botanic extract of gum mastic, showed some encouraging results in improving visual function in NA-AION patients [154];
***h.***  ***Hyperbaric oxygen therapy (HBO2)****:* the inhalation of pure oxygen during HBO2 is supposed to improve oxygen transport to damaged tissues, which may help the recovery of ischemic ON axons. Furthermore, in experimental animal models of NA-AION, HBO2 has been demonstrated to exert a neuroprotective effect by downregulating the expression of apoptosis-related genes [155]. Although there is conflicting evidence about its efficacy in NA-AION patients, several case reports showing promising results have been published [155];
***i.***  ***Endothelin receptor antagonist***: the bosetan is an endothelin receptor antagonist that has been demonstrated to increase ONH blood flow in healthy and glaucomatous subjects. A multicenter double-blind randomized controlled trial investigating the effect of the oral administration of bosetan during the acute stage of NA-AION is actually in course [156];
- ***Low vision rehabilitation***: may be beneficial in cases of severe visual loss to maximize the patient’s residual eyesight. This might involve training on how to adjust to the visual field defect, the use of magnifying glasses, and visual assistance [157].

### 9.3. Prophylactic Measures: The Individuation and Treatment of Risk Factors and Associated Comorbidities

Considering that no available effective treatment of NA-AION exists [1,2,3,4,5,6], the purpose of the actual therapeutic strategies is to identify and possibly control any underlying modifiable risk factors, aiming to likely prevent the development of new NA-AION episodes in the affected and fellow eye [11,45,148,149].

In this regard, ophthalmologists should remember the following important points:

- a complete anamnesis should include [14,44]: past medical history (e.g., vasculopathy risk factors); recent surgical history; social history (e.g., smoking); medications (e.g., amiodarone, PDE-5i); and the presence of symptoms of OSAS and giant cell arteritis, such as daytime sleepiness, pain, mandibular claudication, etc.;
- invite all NA-AION patients to undergo a complete multidisciplinary evaluation by an inter-professional team, including internists, endocrinologists, neurologists, and pulmonologists to identify and likely control modifiable risk factors such as systemic hypertension, diabetes mellitus, hyperlipidemia, and OSAS;
- request all NA-AION patients to stop smoking, reduce weight, and do physical exercise to reduce the risk of vascular diseases that are linked to NA-AION development [14,58,62];
- avoid nocturnal arterial hypotension, which has been demonstrated to be an important predisposing risk factor for NA-AION. In this regard, avoidance of the assumption of anti-hypertensive drugs in the evening or at bedtime could be important [11];
- ask NA-AION patients to avoid sleeping in the lateral decubitus position, in particular from the site of the affected eye [51];
- suggest ocular hypotensive treatments in the presence of borderline or high IOP values to improve ONH blood flow;
- use caution in prescribing treatment with intravitreal injections in NA-AION patients, because the sudden IOP increase after the intravitreal injection could impair OD circulation and predispose to progressive VA loss in the affected eye, or the development of NA-AION in the fellow eye [79];
- ask all male patients developing an NA-AION about the use of phosphodiesterase-5 inhibitors (PDI5i); moreover, before the prescription of PDI5i, patients should undergo an ophthalmological examination and, especially in the presence of a crowded OD, be informed about the risk of developing an NA-AION by using the drugs and eventually discouraged to take PDE5i. Although the association between NA-AION and the assumption of PDE5i is still debated, several authors suggest that a history of NA-AION should be an absolute contraindication to PDI5i therapy [81,82];
- ask all NA-AION patients about the possible use of amiodarone and, in positive cases, the cardiologist should be alerted about the possible association between the optic neuropathy and assumption of the drug [84];
- inform all female NA-AION patients taking oral contraceptives about a possible causal association with the development of NA-AION [85];
- supplementation with vitamin B6, B12, and folic acid is recommended in the presence of hyperhomocysteinemia, although the value of lowering the homocysteine levels for a reduction in vascular events remain unproven [3].

### 9.4. Future Research and Treatments

- ***Stem cell therapy***: represents a promising therapeutic strategy for NA-AION patients. Research on the use of stem cells to support optic nerve regeneration and repair is ongoing, and preliminary findings in animal models are encouraging. The development of a stem cell therapy for NAION is currently in its early phases in human clinical trials. Stem cells have been demonstrated to have the potential of ischemic neural tissue damage repair via promoting angiogenesis, neuro-regeneration, and neuro-recovery, reducing apoptosis, and suppressing oxidative stress and inflammation. Moreover, stem cells are considered a viable option for generating new RGCs as a result of neuronal trans-differentiation. The retrobulbar, subtenon, and intravenous administration of bone-marrow-derived stem cells in a small cohort of NA-AION patients showed a significant improvement in VA within six months post-procedure in 80% of treated eyes [158]. The results of the intravitreal injection of autologous mesenchymal stem cell exosome in NA-AION patients are currently under investigation [159];
- ***Gene therapy***: the use of gene therapy to encourage ON regeneration and repair is another field of research. Gene therapy has been investigated to replace gene mutations in disorders affecting the ON and to alter genes that suppress or activate pathways of ON growth and regeneration. Animal studies using gene therapy have demonstrated some degree of ON axon regeneration and RGCs apoptosis reduction [160]. Researchers are investigating how to transmit genes that can promote neuron development and repair using viral vectors. To ascertain the safety and effectiveness of this strategy, additional research is required as it is currently in the experimental stage.

## 10. Summary

Non-arteritic anterior ischemic optic neuropathy is a common disease with serious consequences for patients, representing one of the most important causes of blindness or severely impaired vision in middle-aged and elderly people [10].

The distinctive clinical presentation of NA-AION, that allows for its clinical diagnosis, is an acute unilateral painless visual loss associated with an OD swelling [1,2,3,4,5,6,11,45].

The exact etiology and pathophysiology of NA-AION are not yet completely understood and still highly discussed, and the paucity of NA-AION cases examined histologically contributes to an incomplete understanding of its pathogenesis [16]. It is commonly accepted that NA-AION is caused by an acute deficit in ONH blood-flow supply, resulting in an acute ischemic infarction of the ON, with successive apoptosis of the retinal ganglion cells [1,7,8,9,17].

More precisely, it is supposed that transient or permanent hypoperfusion of the short posterior ciliary arteries, that supply the ONH, may induce an OD edema that could incite a compartment syndrome in a non-expansible region—the site between the ONH surface and the lamina cribrosa—especially in structurally predisposed crowded ODs. A secondary inflammation could magnify the damage to the ON axons and glial tissue, with, finally, secondary RGCs death by apoptosis [1,7,8,9,17]. This sequence of events has been confirmed in animal (rodent and non-human primate) models of NA-AION obtained by laser-induced damage of the ONH capillaries [40]. However, experimental results need to be interpreted with caution due to the anatomical and pathophysiological differences amongst species [17].

Although a precise major cause is not found in the majority of cases, several precipitating variables have been suggested to explain the occurrence of OD acute circulatory insufficiency, including generalized hypoperfusion, nocturnal hypotension, vasospasm, local arteriosclerosis vascular occlusion, venous occlusion, thrombosis, embolization, or multiple micro-embolism embolisms from a remote source [7,8,9,17]. Moreover, it is thought that the presence of an impaired vascular autoregulation mechanism at the ONH level could be an important favoring factor in NA-AION development [7].

Several other predisposing or precipitating risk factors and comorbidities have been associated with NA-AION onset, including a mix of anatomical abnormalities, genetic susceptibility, systemic vascular risk factors, and pharmaceutical use [1,3,5,11,12,14,44]. The presence of different risk factors in different patients has prognostic and therapeutic implications. A better comprehension of the underlying pathophysiological mechanisms and risk factors of NAION is crucial to identify people at a higher risk of developing the disease and to apply preventive measures and early intervention to stop irreversible vision loss.

NA-AION diagnosis is primarily clinical, and is based on clinical presentation, fundus examination, the patient’s medical history, and the exclusion of other possible etiologies [1,2,4,110]. Bearing in mind that a visual field defect is always present at onset, whereas a visual acuity loss can be absent in one third of cases, perimetry is considered to be the most important diagnostic test in NA-AION [11,103].

It is important to differentiate NA-AION from other pathologies for both prognostic and therapeutic reasons.

Considering that other causes of OD swelling include sight- and life-threatening diseases, including arteritic AION (A-AION), demyelinating, infiltrative, and compressive optic neuropathies [110,111], early diagnosis is crucial to prevent irreversible vision loss and enhance patient outcomes. In particular, when ischemic optic neuropathy is suspected, the exclusion of the arteritic form due to giant cell arteritis (GCA) is mandatory. GCA represents, indeed, a serious vision- and life-threatening condition and it could be considered the only real medical emergency in ophthalmology because its early diagnosis and treatment with high-dose steroid therapy may prevent blindness and severe systemic complications [46,47]. A differential diagnosis may require perimetry, blood exams, fluorescein angiography, OCT and OCT-angiography, and RMN of the brain and orbits.

NA-AION has a relatively bad prognosis: in the majority of cases, it results in permanent vision loss, with 40–50% of patients having significant visual impairment or being legally blind in the affected eye [2].

The risk of recurrence in the same eye has been estimated to be 3–8% [129,133], whereas the involvement of the fellow eye has been reported to occur in 15–30% of cases within five years of the first eye damage [99]. Although numerous medical treatments and procedures have been suggested and tried [1,11,45,110,148,149], no effective treatments or prophylactic measures for NA-AION have been found at present [2,3,4,5,6]. Considering that NA-AION seems to be a multifactorial disease, the existence of a general therapeutic option seems to be unlikely, because different etiologies may probably require different treatment approaches. The purpose of actual therapeutic strategies is therefore to identify and possibly control any underlying modifiable risk factors and associated comorbidities [14,44], aiming to likely prevent the development of new NA-AION episodes in the affected and fellow eye [3,5,45]. The NA-AION patient’s care may require the involvement of a multi-professional team composed of general physicians, endocrinologists, neurologists, and pulmonologists to improve communication and therapeutic coordination; close observation and frequent follow-ups are also mandatory.

Numerous prospective medicines are being researched in the continuous hunt for effective NA-AION treatments and there is promise for future improvements in the treatment of this illness: animal models of NA-AION have been developed to better understand the pathophysiology of this disease and to provide a platform to evaluate new therapies [40]; moreover, current research into prospective remedies such as neuroprotective drugs, stem cell therapy, and gene therapy aiming to stop or reduce the optic nerve damage in NA-AION, are on course [148,149,160].

## 11. Conclusions

In conclusion, this review provides a thorough and invaluable resource for both healthcare professionals and individuals seeking to understand the complexities of NA-AION. By delving into the epidemiology, risk factors, clinical presentation, diagnostic approaches, and management strategies, clinicians can be equipped with an important understanding of this challenging optic neuropathy. Furthermore, it underscores the importance of ongoing research and collaboration in the pursuit of better treatments and preventive measures. As the medical community continues to refine its knowledge of NA-AION, future studies are needed to provide a brighter future for those affected by this condition.

## Figures and Tables

**Table 1:** Typical and atypical clinical features of NA-AION.

Signs or Symptoms	Typical	Atypical
**Patient’s age**	over 50 years	less than 50 years
**Onset of visual loss**	acute	rapidly sequential or gradually slowly progressive
**Onset laterality**	unilateral	bilateral simultaneous or sequential
**Visual field defects**	altitudinal or arcuate	central scotoma, homonymous hemianopsia, etc.
**OD edema**	segmental, regressing within 6–11 weeks	hyperemic, chalky white, persisting more than 6–11 weeks
**OD the fellow eye at onset**	small C/D	normal or large C/D
**Peripapillary hemorrhages**	common	uncommon
**Relative afferent pupillary defect**	present	absent
**Ocular pain**	absent	present, worsening with eye movements
**Premonitory symptoms**	uncommon	common (amaurosis fugax, diplopia)
**Associated ocular finding**	OD drusen, hypertensive or diabetic retinopathy	macular star, proptosis, lid or eye movement abnormalities
**Presence of vasculopatic risk factors**	common	absent
**Associated systemic symptoms**	absent	fever, malaise, jaw claudication, headache, abnormal temporal artery
**Disease course**	stabilization within two weeks	recurrent attacks
**Response to CS therapy**	unclear	present
**MRI brain and orbit findings**	none	optic nerve enhancement or sheath

OD = optic disk; C/D = cup-to-disk ratio; CS = corticosteroids.

**Table 2:** Differential diagnosis amongst NA-AION, A-AION, optic neuritis, anterior orbital lesions.

Clinical Features	NA-AION	A-AION	Papillitis	Orbital Lesions
**Patient’s age**	any, most frequent over 50 years	over 50 years, most frequent over 70 years	any, most common in young	any
**Gender predilection**	male	female	female	any
**Visual loss onset**	acute	acute, poor VA *	semi-acute	gradually slowly progressive
**Onset laterality**	unilateral	unilateral or bilateral (30% of cases)	unilateral	unilateral
**Visual field defect pattern**	altitudinal or arcuate	altitudinal or arcuate	central, centro-cecal, arcuate	arcuate, peripheral
**OD edema**	any type, segmental, evolution to OD atrophy	chalky white, segmental, evolution to OD cupping	mild, hyperemic, evolution to OD atrophy	pale, lasting over 4–6 weeks, evolution to OD atrophy
**OD the fellow eye at onset**	small C/D	normal or large C/D	normal or large C/D	normal or large C/D
**Peripapillary hemorrhages**	common	common	uncommon	uncommon
**Relative afferent pupillary defect**	present	present	present	present
**Ocular pain**	uncommon (10–15% of cases)	common (75% of cases)	common (95% of cases), worse with eye movements	common
**Premonitory symptoms**	uncommon	amaurosis fugax and/or diplopia (30% of cases)	uncommon	uncommon
**Associated ocular finding**	OD drusen, hypertensive or diabetic retinopathy	cotton wool spots, central retinal o cilioretinal artery occlusion	possible intraocular inflammation, retinal vasculitis	ptosis, proptosis, lid and eye movements abnormalities
**Presence of vasculopatic risk factors**	common	common	uncommon	uncommon
**Associated systemic symptoms**	absent	fever, malaise, jaw claudication, headache, abnormal temporal artery **	paresthesia, diplopia, ataxia, weakness, systemic disease	signs of systemic malignancy may be present
**Disease course**	frequent spontaneous visual recovery	rare spontaneous visual recovery, severe prognosis	frequent spontaneous visual recovery	progressively worse
**FAG features of OD ischemia**	common	common and severe	absent	absent
**serous ESE and CRP levels**	normal	significantly high	normal	normal
**MRI brain and orbit findings**	none	ON sheath and orbital fat enhancement	ON enhancement; frequent signs of demyelination	presence of orbital compressive lesions
**Response to CS therapy**	unclear	present, stop of further vision loss and systemic complications	present, with good visual prognosis	absent

NA-AION = non-arteritic anterior ischemic optic neuropathy; A-AION = arteritic anterior ischemic optic neuropathy; OD = optic disk; C/D = cup-to-disk ratio; ON = optic nerve; CS = corticosteroids; CRP = C-reactive protein; * = VA at onset ≤ counter finger in >50% of cases; ** = 25% of A-AION are “occult”, i.e., without systemic symptoms.

## Abbreviations

ION	ischemic optic neuropathy
NA-AION	non-arteritic anterior ischemic optic neuropathy
A-AION	arteritic anterior ischemic optic neuropathy
OD	optic disc
ON	optic nerve
ONH	optic nerve head
RGC	retinal ganglion cell
VA	visual acuity
VF	visual field
IOP	intraocular pressure
RNFL	retinal nerve fiber layer
RAPD	relative afferent pupillary defect
